# The economic impact of cannabis use disorder and dementia diagnosis in veterans diagnosed with traumatic brain injury

**DOI:** 10.3389/fneur.2023.1261144

**Published:** 2024-01-12

**Authors:** Aryan Esmaeili, Terri K. Pogoda, Megan E. Amuan, Carla Garcia, Ariana Del Negro, Maddy Myers, Mary Jo Pugh, David Cifu, Clara Dismuke-Greer

**Affiliations:** ^1^Health Economics Resource Center (HERC), Ci2i, VA Palo Alto Health Care System, Menlo Park, CA, United States; ^2^Center for Healthcare Organization and Implementation Research, VA Boston Healthcare System, Boston, MA, United States; ^3^Boston University School of Public Health, Boston, MA, United States; ^4^Informatics, Decision-Enhancement, and Analytic Sciences Center of Innovation, VA Salt Lake City Health Care System, Salt Lake City, UT, United States; ^5^Division of Epidemiology, Department of Internal Medicine, University of Utah School of Medicine, Salt Lake City, UT, United States; ^6^Department of Physical Medicine and Rehabilitation, School of Medicine, Virginia Commonwealth University, Richmond, VA, United States

**Keywords:** traumatic brain injury, cannabis use disorder, dementia, veterans, costs, economic burden, healthcare utilization

## Abstract

**Background:**

Studies have demonstrated that individuals diagnosed with traumatic brain injury (TBI) frequently use medical and recreational cannabis to treat persistent symptoms of TBI, such as chronic pain and sleep disturbances, which can lead to cannabis use disorder (CUD). We aimed to determine the Veterans Health Administration (VHA) healthcare utilization and costs associated with CUD and dementia diagnosis in veterans with TBI.

**Methods:**

This observational study used administrative datasets from the population of post-9/11 veterans from the Long-term Impact of Military-Relevant Brain Injury Consortium-Chronic Effects of Neurotrauma Consortium and the VA Data Warehouse. We compared the differential VHA costs among the following cohorts of veterans: (1) No dementia diagnosis and No CUD group, (2) Dementia diagnosis only (Dementia only), (3) CUD only, and (4) comorbid dementia diagnosis and CUD (Dementia and CUD). Generalized estimating equations and negative binomial regression models were used to estimate total annual costs (inflation-adjusted) and the incidence rate of healthcare utilization, respectively, by dementia diagnosis and CUD status.

**Results:**

Data from 387,770 veterans with TBI (88.4% men; median [interquartile range (IQR)] age at the time of TBI: 30 [14] years; 63.5% white) were followed from 2000 to 2020. Overall, we observed a trend of gradually increasing healthcare costs 5 years after TBI onset. Interestingly, in this cohort of veterans within 5 years of TBI, we observed substantial healthcare costs in the Dementia only group (peak = $46,808) that were not observed in the CUD and dementia group. Relative to those without either condition, the annual total VHA costs were $3,368 higher in the CUD only group, while no significant differences were observed in the Dementia only and Dementia and CUD groups.

**Discussion:**

The findings suggest that those in the Dementia only group might be getting their healthcare needs met more quickly and within 5 years of TBI diagnosis, whereas veterans in the Dementia and CUD group are not receiving early care, resulting in higher long-term healthcare costs. Further investigations should examine what impact the timing of dementia and CUD diagnoses have on specific categories of inpatient and outpatient care in VA and community care facilities.

## Introduction

Acute traumatic brain injury (TBI) is associated with a decline in cognition involving one or more domains (learning and memory, language, executive function, complex attention, perceptual-motor, and social cognition) (1), and TBI-related symptoms may persist for more than 6 months post-injury (2). The economic impact of TBI on the United States Department of Veterans Affairs (VA) has been shown to extend over a decade (≥15 years) (3); however, TBI-related costs in the Department of Defense (DOD) or VA may even be underestimated compared with the civilian sector considering that service members and veterans face a higher risk of TBI within their lifetime (4) and are more likely to suffer from injury-related comorbidities, such as chronic pain, post-traumatic stress disorder (PTSD), and other mental health conditions (5–8). Up to 20% of more than 2.5 million deployed service members since 2003 are estimated to have sustained at least one TBI (9, 10). Comorbid mental health diagnosis, substance use disorders, general medical disorders, TBI, history of violent events, and suicide attempts increase the risk of post-injury recurrent hospitalizations and deaths (8).

Mild cognitive impairment prevalence in the US is 6.7% for ages 60–64 years, 8.4% for 65–69 years, and increases to 25.2% for 80–84 years (11). Dementia, a disease of older age, has an overall prevalence of 7.3% among the VA healthcare system users older than 65 years old (12). However, TBI is considered a risk factor for dementia diagnosis (13) and early onset dementia in veterans, defined as dementia onset in age less than 65 years (14). Veterans with comorbid TBI and dementia have a higher healthcare cost burden relative to TBI alone or those with no diagnosis, and older veterans with comorbid TBI and dementia have been shown to have higher annual total Veteran Health Administration (VHA) costs (3).

Individuals with TBI who suffer from chronic pain are at a higher risk of substance and/or opioid use disorder (OUD) (15), and the presence of a TBI history should be considered in clinical decision-making regarding the long-term use of opioids (16, 17). Approximately 23% of individuals with OUD appear to also use cannabis (18). Despite the lack of proven efficacy, cannabis is frequently used to self-treat a wide array of symptoms and conditions associated with post-TBI injury (e.g., chronic pain, headache, sleep disturbances, anxiety, and irritability) (19–21). Cannabis use disorder (CUD) presents as a problematic pattern of cannabis use, with either abuse or dependence, that results in clinically significant functional impairment or distress. Following the cannabis legalization process, similar to the general population, access to cannabis and cannabinoids has increased substantially among VA patients (22). The estimated prevalence of cannabis use in veterans increased from 9% in 2014 to nearly 12% in 2020 (23); however, its efficacy and safety profile remains uncertain (24, 25). While the antioxidant and anti-inflammatory properties of cannabidiol suggest protective effects of cannabis on dementia progression (26), dementia-like structural changes to the brain have also been observed in heavy, chronic cannabis users (27–31).

Dementia is a possible long-term comorbidity of TBI, potentially accelerated by the presence of chronic pain, and secondary substance and cannabis use may have a specific, critical role in the dementia process after TBI. All of these factors individually and in combination are likely to have overlapping and additive health effects that necessitate the availability and use of general and targeted VA healthcare resources. This study aimed to determine the VA healthcare costs associated with CUD and dementia diagnosis in veterans with TBI.

## Methods

### Participants and data source

Our cohort included participants from the Long-term Impact of Military-Relevant Brain Injury Consortium–Chronic Effects of Neurotrauma Consortium (LIMBIC-CENC) phenotype study, which has been described in detail previously (32). The LIMBIC phenotype longitudinal cohort is a large cohort of post-9/11 (including Operation Enduring Freedom and Operation Iraqi Freedom) current and former US military persons who received care in the DoD for at least 3 years, including those exposed and unexposed to TBI(s). The goal of this cohort is to identify chronic sequelae and neurologic comorbidities (cognitive, behavioral, and physical). Sources for this study included healthcare data during deployment (e.g., DoD Trauma Registry [DoDTR] and Theatre Management Data Store [TMDS]), DoD, VA, and non-VA community inpatient and outpatient data.

To ensure accurate TBI status and sufficient data to identify dementia, we included all the participants who enrolled in the Veterans Health Administration (VHA) during the study period, completed the TBI screening, and underwent the VA comprehensive TBI evaluation (CTBIE). The LIMBIC-CENC consortium verified the definition for the TBI severity categories. We used a hierarchical approach to identify TBI and its severity by prioritizing data from DoDTR and TMDS, followed by self-reported data from the CTBIE data collected in the process of clinical care, in the alteration of consciousness or post-traumatic amnesia, and according to ICD-9/10-CM diagnosis codes from the 2012 Armed Forces Health Surveillance System algorithm (33, 35, 36). We also considered ICD codes for post-concussive syndrome as evidence of mild TBI history among veterans without another indicator of a TBI diagnosis. The veterans who did not enroll in VHA and did not complete the initial VA screening for CTBIE were excluded from the study. The index date for TBI was the first date of diagnosis or the date of the CTBIE assessment; for those with more than one TBI documented, we used the date of the most severe TBI. The research protocol was reviewed and approved by the institutional review boards of the University of Utah and Stanford and was conducted in accordance with all applicable federal regulations.

### Measures and outcomes

#### VA health services costs

Annual per veteran total costs for both VA and non-VA facilities were obtained for fiscal years 2000 through 2020, the last available year for VA cost data. VA national costs are estimated by the Health Economics Resource Center using actual cost data from VA facilities, including adjustments for labor cost differentials across regions (34). Non-VA facility costs were based on reimbursement by VA to non-VA facilities. In our study, the immediate healthcare costs after acute TBI, which may have been paid by the DoD while the veterans were in service, are not captured. All cost data were adjusted for inflation to 2022-dollar values (37).

Dementia diagnosis was identified using ICD-9/10 codes provided by VHA geriatrics and extended care (Supplementary Table S1). ICD 9/10 codes used to identify dementia in older patients have been found to be inaccurate when used in patients under the age of 65 years (38, 39). CUD was identified using ICD-9 (304.3: Cannabis dependence, and 305.2: Nondependent cannabis abuse) and ICD-10 codes (F12: Cannabis-related disorders). We compared the differential VHA costs among the following groups of veterans with a history of TBI: (1) No Dementia diagnosis and No CUD (control group), (2) Dementia diagnosis only (Dementia only), (3) CUD only, and (4) comorbid dementia diagnosis and CUD (Dementia and CUD).

We used a quality-cost conceptual framework to select the covariates and risk factors associated with TBI, dementia, and CUD health services costs (40). The sociodemographic and military characteristics (sex, age at TBI diagnosis (baseline), race, education, marital status, branch, rank, rurality, VA service-connected disability percent, and district/region) were obtained from the VA and DoD Identity Repository (VADIR). Years of TBI diagnosis were captured by the total number of years since the first TBI diagnosis and enrolled in VHA. Other covariates were defined using ICD-9/10 codes from VINCI and DOD VA Informatics and Computing Infrastructure (DaVINCI) and are defined in Supplementary Table S1. These measured conditions have a complex multifactorial etiology and are risk factors for dementia, CUD, and TBI (14, 41, 42).

### Statistical analysis

We conducted descriptive analyses of demographic characteristics and risk behaviors from baseline data by CUD and dementia diagnosis status. To evaluate the healthcare cost trajectories over time by dementia and CUD status, we plotted the annual total costs after the TBI index date. We have also presented the trajectories of the dementia and CUD cost stratified by TBI severity. We assessed the association between total healthcare costs and CUD or dementia diagnosis status and the 95% confidence interval (CI), using crude and adjusted generalized estimating equation (GEE) models. The incidence rate ratio of healthcare utilization by CUD or dementia diagnosis status was reported using a negative binomial regression model. The following covariates in the adjusted model included sociodemographic and military characteristics (years of TBI diagnosis, biological sex, age at the time of TBI, TBI severity, race/ethnicity, highest education level completed at baseline, marital status, military branch, rank, rurality, service-connected disability percentage, US district (region), and death), and health conditions (see more details in Supplementary Table S1). We repeated the GEE-adjusted model for veterans with at least two dementia diagnosis codes for a sensitivity analysis. The association between healthcare costs and utilization and TBI severity in these adjusted models is also reported. Using the standardized mean difference, the risk profile of dementia and CUD have been separately evaluated, and we report the clinical and structural population differences for measured covariates in Supplementary Table S2 (43, 44). All analyses were conducted using Stata version 17 (StataCorp LP, College Station, Texas).

## Results

### Sociodemographic/military and clinical characteristics

**Figure 1:**
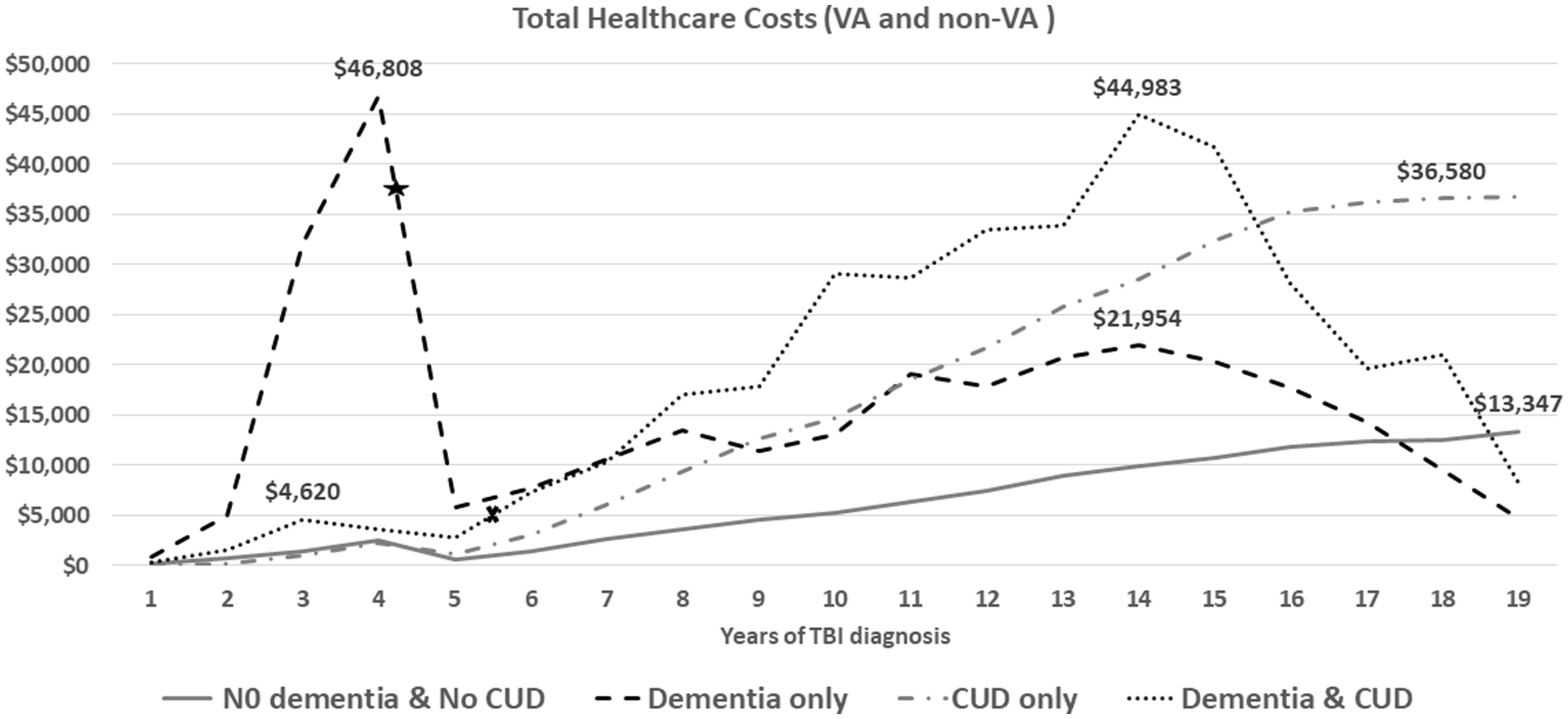
The average of annual total health care costs (VA and non-VA) after TBI injury (time zero). The average time from TBI event to dementia diagnosis was 4.36 years for veterans in the dementia only (star) and 5.31 years for veterans in the dementia and CUD group (x).

Table 1 presents the demographic and medical conditions characteristics for four groups of veterans diagnosed with TBI: (1) No Dementia diagnosis and No CUD group (*n* = 341,324; 88.02%), (2) Dementia only (*n* = 4,572;1.18%), (3) CUD only (*n* = 40,873; 10.54%), and (4) Dementia and CUD (*n* = 1,001; 0.26%). The median [interquartile range] age at the time of TBI was 30 [14] years. The majority of the veterans (65.54%) in our cohort presented with mild TBI (Table 1). Veterans diagnosed with TBI who were diagnosed with dementia and CUD were predominantly non-Hispanic white people, men, and former army service members with up to a high school education. They had relatively high rates of non-headache chronic pain and insomnia as well as severe mental illness and other mental health diagnoses such as depression, anxiety, and personality disorders. They also had relatively high rates of alcohol use disorder and OUD. The clinical and structural population differences for measured covariates indicate substantial differences between dementia and non-dementia as well as the CUD and non-CUD groups (see Supplementary Table S2). The time from TBI to dementia was approximately 1 year longer in the Dementia and CUD group (mean (SD) = 4.36 (4.18) years) compared with the Dementia only group (mean (SD) = 5.31 (4.10) years), as shown in Figure 1.

**Table 1:** Demographic and clinical characteristics of veterans with a history of TBI by dementia diagnosis and CUD status (*N* = 387,770).

	No Dementia and No CUD *N* (%)	Dementia only *N* (%)	CUD only *N* (%)	Dementia and CUD *N* (%)	Total *N* (%)
Overall	341,324 (88.02)	4,572 (1.18)	40,873 (10.54)	1,001 (0.26)	387,770 (100.00)
Male sex	300,291 (87.98)	4,078 (89.2)	37,319 (91.3)	924 (92.31)	342,612 (88.35)
Age ≥ 65	2,228 (0.65)	316 (6.91)	27 (0.07)	4 (0.40)	2,575 (0.66)
*Race and ethnicity*					
White	217,674 (63.77)	2,887 (63.15)	25,167 (61.57)	629 (62.84)	246,357 (63.53)
Black/African American	54,081 (15.84)	800 (17.5)	7,042 (17.23)	169 (16.88)	62,092 (16.01)
Hispanic or Latino	35,339 (10.35)	432 (9.45)	3,543 (8.67)	86 (8.59)	39,400 (10.16)
Other	32,957 (9.66)	446 (9.76)	5,036 (12.32)	114 (11.39)	38,553 (9.94)
Unknown	1,273 (0.37)	7 (0.15)	85 (0.21)	3 (0.30)	1,368 (0.35)
*Education*					
College and above	81,834 (23.98)	1,679 (36.72)	4,349 (10.64)	142 (14.19)	88,004 (22.69)
High school and less	258,910 (75.85)	2,873 (62.84)	36,474 (89.24)	856 (85.51)	299,113 (77.14)
Unknown	580 (0.17)	20 (0.44)	50 (0.12)	3 (0.30)	653 (0.17)
*Marital status*					
Unmarried	161,077 (47.19)	1,763 (38.56)	26,386 (64.56)	614 (61.34)	189,840 (48.96)
Married	180,067 (52.76)	2,808 (61.42)	14,471 (35.4)	386 (38.56)	197,732 (50.99)
Unknown	180 (0.05)	1 (0.02)	16 (0.04)	1 (0.1)	198 (0.05)
*Military branch*					
Air Force	33,398 (9.78)	686 (15)	2,399 (5.87)	88 (8.79)	36,571 (9.43)
Army	204,047 (59.78)	2,615 (57.2)	27,442 (67.14)	658 (65.73)	234,762 (60.54)
Marines	61,003 (17.87)	551 (12.05)	6,822 (16.69)	135 (13.49)	68,511 (17.67)
Navy/Coast guard	42,725 (12.52)	708 (15.49)	4,208 (10.3)	120 (11.99)	47,761 (12.32)
Other	151 (0.04)	12 (0.26)	2 (0)	0 (0)	165 (0.04)
*Rank*					
Enlisted	317,218 (92.95)	3,916 (85.65)	40,288 (98.57)	974 (97.3)	362,396 (93.46)
Officer	20,407 (5.98)	562 (12.29)	484 (1.18)	24 (2.4)	21,477 (5.54)
Warrant	3,670 (1.08)	94 (2.06)	99 (0.24)	3 (0.3)	3,866 (1)
*Rurality*					
Rural	108,255 (31.72)	1,455 (31.82)	12,632 (30.91)	309 (30.87)	122,651 (31.63)
Urban	231,902 (67.94)	3,109 (68)	28,151 (68.87)	689 (68.83)	263,851 (68.04)
Unknown	1,167 (0.34)	8 (0.17)	90 (0.22)	3 (0.3)	1,268 (0.33)
VA SCD% (0)	38,954 (11.41)	665 (14.55)	4,928 (12.06)	132 (13.19)	44,679 (11.52)
10 to 40%	24,126 (7.07)	157 (3.43)	1,821 (4.46)	10 (1)	26,114 (6.73)
≥50%	278,244 (81.52)	3,750 (82.02)	34,124 (83.49)	859 (85.81)	316,977 (81.74)
*District*					
North Atlantic	70,682 (20.71)	956 (20.91)	8,177 (20.01)	188 (18.78)	80,003 (20.63)
Southeast	66,029 (19.35)	1,090 (23.84)	7,634 (18.68)	198 (19.78)	74,951 (19.33)
Midwest	69,456 (20.35)	854 (18.68)	8,524 (20.85)	177 (17.68)	79,011 (20.38)
Continental	72,831 (21.34)	1,042 (22.79)	8,722 (21.34)	260 (25.97)	82,855 (21.37)
Pacific	62,318 (18.26)	630 (13.78)	7,816 (19.12)	178 (17.78)	70,942 (18.3)
*Comorbid conditions*					
Headache	194,574 (57.01)	3,178 (69.51)	24,497 (59.93)	729 (72.83)	222,978 (57.5)
Other chronic pain	309,908 (90.8)	4,386 (95.93)	38,233 (93.54)	964 (96.3)	353,491 (91.16)
MAT (recent)	18,957 (5.55)	335 (7.33)	11,333 (27.73)	317 (31.67)	30,942 (7.98)
Oncology	4,257 (1.25)	152 (3.32)	611 (1.49)	20 (2)	5,040 (1.3)
SMI	80,657 (23.63)	2,184 (47.77)	24,516 (59.98)	786 (78.52)	108,143 (27.89)
Depression	187,134 (54.83)	3,614 (79.05)	34,257 (83.81)	940 (93.91)	225,945 (58.27)
PTSD	222,236 (65.11)	3,058 (66.89)	35,512 (86.88)	895 (89.41)	261,701 (67.49)
Personality disorder	15,343 (4.5)	474 (10.37)	9,748 (23.85)	375 (37.46)	25,940 (6.69)
Alcohol use disorder	120,007 (35.16)	1,967 (43.02)	33,786 (82.66)	886 (88.51)	156,646 (40.4)
OUD	20,292 (5.95)	532 (11.64)	18,187 (44.5)	533 (53.25)	39,544 (10.2)
Other drug use disorder	23,526 (6.89)	624 (13.65)	26,766 (65.49)	790 (78.92)	51,706 (13.33)
Nicotine use disorder	91,915 (26.93)	1,494 (32.68)	23,006 (56.29)	676 (67.53)	117,091 (30.2)
Anxiety	171,582 (50.27)	3,098 (67.76)	30,976 (75.79)	867 (86.61)	206,523 (53.26)
Insomnia	114,882 (33.66)	2,246 (49.13)	16,147 (39.51)	557 (55.64)	133,832 (34.51)
CHF	6,880 (2.02)	504 (11.02)	794 (1.94)	70 (6.99)	8,248 (2.13)
Peripheral vascular disease	12,441 (3.64)	840 (18.37)	1,152 (2.82)	93 (9.29)	14,526 (3.75)
Cardiac disease	48,580 (14.23)	1,618 (35.39)	7,806 (19.1)	372 (37.16)	58,376 (15.05)
Stroke	12,634 (3.7)	1,145 (25.04)	1,538 (3.76)	187 (18.68)	15,504 (4)
DM	37,526 (10.99)	1,219 (26.66)	2,985 (7.3)	178 (17.78)	41,908 (10.81)
Diabetes with chronic complication	21,038 (6.16)	795 (17.39)	1,600 (3.91)	99 (9.89)	23,532 (6.07)
Epilepsy	85,489 (25.05)	2,186 (47.81)	19,406 (47.48)	664 (66.33)	107,745 (27.79)
Other neurologic disorders (no epilepsy)	6,515 (1.91)	889 (19.44)	992 (2.43)	139 (13.89)	8,535 (2.2)
Liver disease	12,905 (3.78)	325 (7.11)	1,810 (4.43)	83 (8.29)	15,123 (3.9)
CKD	6,233 (1.83)	302 (6.61)	801 (1.96)	43 (4.3)	7,379 (1.9)
Death	10,068 (2.95)	647 (14.15)	2,561 (6.27)	119 (11.89)	13,395 (3.45)
*TBI severity and evidence of TBI*					
Mild	223,940 (65.61)	2,304 (50.39)	27,358 (66.93)	539 (53.85)	254,141 (65.54)
Moderate/Severe	44,421 (13.01)	955 (20.89)	6,069 (14.85)	233 (23.28)	51,678 (13.33)
Penetrating	11,582 (3.39)	702 (15.35)	1,450 (3.55)	137 (13.69)	13,871 (3.58)
Unclassified	61,381 (17.98)	611 (13.36)	5,996 (14.67)	92 (9.19)	68,080 (17.56)

CUD, cannabis use disorder; SCD, service-connected disability; TBI, traumatic brain injury; MAT, medication-assisted treatment; CHF, congestive heart failure; CKD, chronic kidney disease; PTSD, post traumatic stress disorder; SMI, severe mental illness; DM, diabetes mellitus.

### Healthcare (VA and non-VA) costs after TBI injury

**Figure 2:**
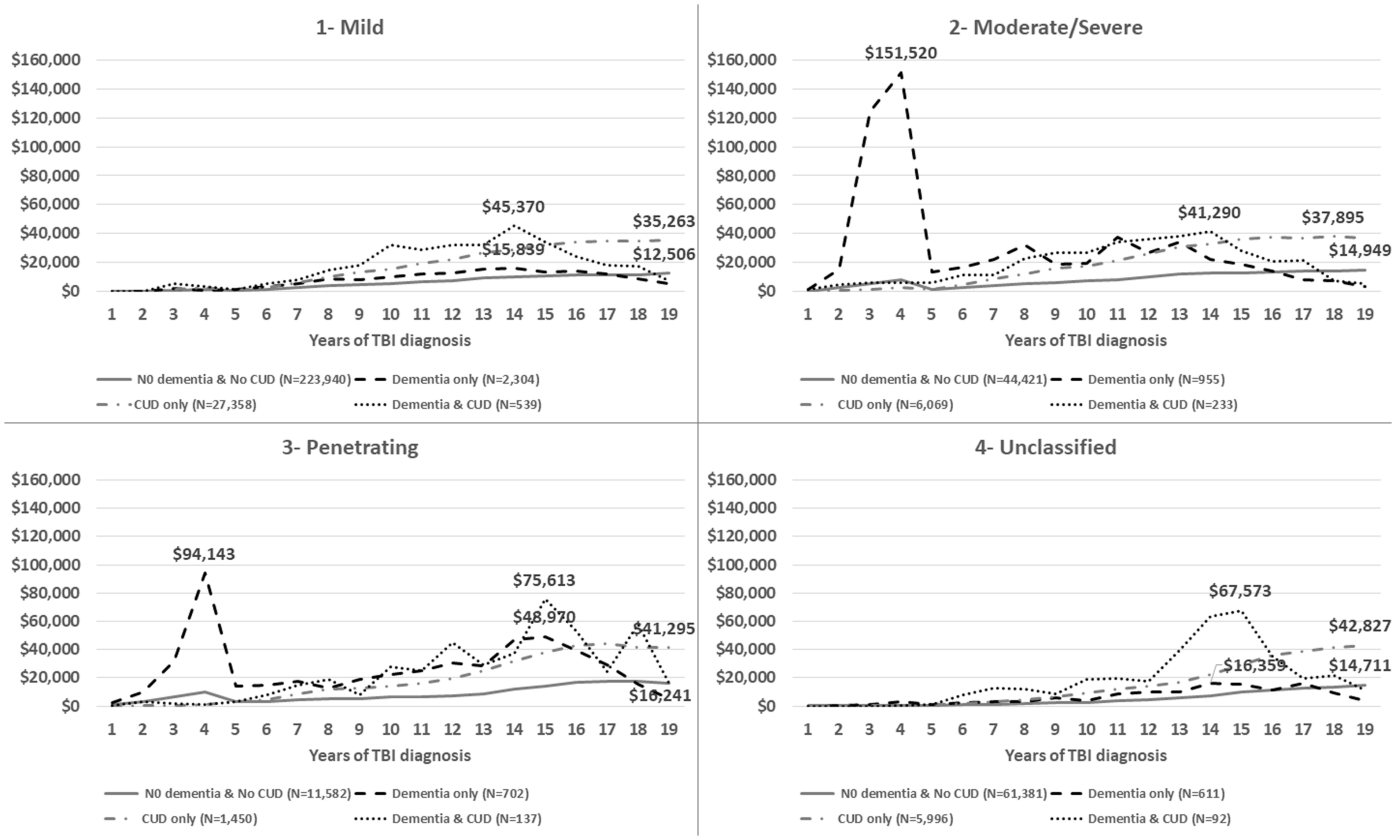
The average of annual total healthcare costs (VA and non-VA) after TBI injury (time zero), stratified by TBI severity (1—mild, 2—moderate/severe, 3—penetrating, 4—post-concussive syndrome, 5—unclassified).

Figure 1 shows the trend of annual total healthcare costs per veteran after documented TBI over a time span of 19 years. The total costs for the Dementia only and Dementia and CUD groups showed two important trajectories over time (Figure 1). First, we observed substantial healthcare costs in the Dementia only group (peak = $46,808) within 5 years of TBI onset, which was not noticed in other groups (in particular, the Dementia and CUD group). The TBI severity subgroup evaluation showed that the substantial healthcare costs in the Dementia only group were driven by veterans with moderate/severe and penetrating TBI (Figure 2). Second, we observed the gradually increasing trend of healthcare costs after 5 years of TBI onset (Figure 1). However, compared with the two other non-dementia groups, we observed that the increasing trend of healthcare costs in the Dementia and CUD and Dementia only groups declined after approximately 14 years (peak = $44,983 and $21,954 for Dementia and CUD and Dementia only, respectively). We observed a constant increase in the total healthcare costs (VA and non-VA) for the No Dementia and No CUD group and CUD only group, with a higher cost following TBI over time for the CUD only group.

### The association between healthcare costs and CUD and dementia diagnosis status

Table 2 shows the association between healthcare utilization costs and CUD and dementia diagnosis status in veterans with a history of TBI. After controlling for sociodemographic/military characteristics and clinical conditions (Model 2), the total healthcare costs were USD$ 3,368 higher in the CUD only group (95% CI: 3,090–3,645) than in the No Dementia and No CUD group. We did not observe any association between the annualized total healthcare costs in dementia-related subgroups (Dementia only and Dementia and CUD groups), compared with the No Dementia and No CUD group.

**Table 2:** The association between healthcare costs or utilization and CUD and dementia diagnosis status in veterans with a history of TBI.

	Crude regression (Model 1)		Adjusted model (Model 2)		Confirmed dementia Adjusted model (Model 3)	
	Coefficient/IRR (95 CI%)	*p*	Coefficient/IRR (95 CI%)	*p*	Coefficient/IRR (95 CI%)	Value of *p*
*Total healthcare costs, compared with the No Dementia and No CUD group (coefficient)*						
No Dementia and No CUD	Reference		Reference		Reference	
Dementia only	9,294 (4,747, 13,841)	<0.001	1,071 (−3,204, 5,347)	0.623	−344 (−3,023, 2,334)	0.801
CUD only	10,840 (10,597, 11,084)	<0.001	3,368 (3,090, 3,645)	<0.001	3,273 (3,000, 3,545)	<0.001
Dementia and CUD	12,515 (10,753, 14,278)	<0.001	−1,667 (−3,456, 121)	0.068	−116 (−3,109, 2,877)	0.939
*Total healthcare utilizations, compared with No Dementia and No CUD group (IRR)*						
No Dementia and No CUD	Reference		Reference		Reference	
Dementia only	0.36 (0.35, 0.37)	<0.001	0.25 (0.24, 0.25)	<0.001	0.24 (0.24, 0.25)	<0.001
CUD Only	1.12 (1.12, 1.13)	<0.001	0.99 (0.98, 0.99)	<0.001	0.98 (0.98, 0.99)	<0.001
Dementia and CUD	0.38 (0.37, 0.39)	<0.001	0.25 (0.24, 0.26)	<0.001	0.26 (0.25, 0.27)	<0.001

We used the generalized estimating equations model to estimate the healthcare costs by dementia diagnosis or CUD status. We used the negative binomial regression model to estimate the incidence rate ratio of healthcare utilizations by dementia diagnosis or CUD status. The covariates included in the adjusted model: year with TBI, gender, age at the time of TBI, TBI severity, race, education, marital status, branch, rank, rurality, service connected disabilities (percent), district, headache, chronic pain, medication-assisted treatment (recent), oncology, severe mental illness, depression, post-traumatic stress disorder, personality disorder, alcohol use disorder, OUD, other SUD, nicotine use disorder, anxiety, insomnia, congestive heart failure, perivascular disease, cardiac disease, stroke, diabetes mellitus (DM), DM with complications, epilepsy, neurologic disorder (no epilepsy), liver disease, chronic kidney disease, and death. CUD, cannabis use disorder; SCD, service-connected disability; TBI, traumatic brain injury; IRR, incidence rate ratio.

Table 2 also shows the healthcare utilization incidence rate ratio by CUD and dementia diagnosis status in veterans with a history of TBI. Compared with the No Dementia and No CUD group, the total healthcare utilization was lower in the Dementia only [incidence rate ratio (IRR) = 0.25 (CI95%: 0.24, 0.25)], Dementia and CUD [IRR = 0.25 (CI95%: 0.24, 0.26)], and CUD only [IRR = 0.99 (CI95%: 0.98, 0.99)] groups.

### The association between healthcare costs and TBI severity

After controlling for sociodemographic/military characteristics and clinical conditions (Model 2), veterans with penetrating TBI have the highest average annual costs of approximately USD$ 2,600 (95% CI: 1,936–3,265), followed by moderate/severe TBI [USD$ 1,466 (95% CI: 1,032–1,900)] compared with veterans with mild TBI (Table 3). However, compared with mild TBI, veterans with moderate/severe TBI (IRR = 0.91 (95% CI: 0.91–0.92)) have the highest average annual care utilization, followed by the penetrating TBI [IRR = 0.71 (95% CI: 0.71–0.72)].

**Table 3:** The association between healthcare costs or utilization and TBI severity in veterans with a history of TBI.

	Adjusted model (Model 2)		Confirmed dementia adjusted model (Model 3)	
	Coefficient/IRR (CI%95)	*p*	Coefficient/IRR (CI%95)	*p*
*Total healthcare costs (coefficient)*				
Mild	Reference		Reference	
Moderate/Severe	1,466 (1,032, 1,900)	<0.001	1,466 (1,022, 1,910)	<0.001
Penetrating	2,600 (1,936, 3,265)	<0.001	2,622 (1,989, 3,256)	<0.001
Unclassified	294 (156, 432)	<0.001	294 (156, 433)	<0.001
*Total healthcare utilization (IRR)*				
Mild	Reference		Reference	
Moderate/severe	0.91 (0.91, 0.92)	<0.001	0.91 (0.91, 0.91)	<0.001
Penetrating	0.71 (0.71, 0.72)	<0.001	0.7 (0.7, 0.71)	<0.001
Unclassified	0.72 (0.72, 0.72)	<0.001	0.72 (0.72, 0.72)	<0.001

We used the generalized estimating equations model to estimate the healthcare costs by dementia diagnosis or CUD status to provide healthcare costs by TBI severity. We used the negative binomial regression model to estimate the incidence rate ratio of healthcare utilizations by dementia diagnosis or CUD status to provide healthcare utilizations by TBI severity. The covariates included in the adjusted model: year with TBI, gender, age at the time of TBI, race, education, marital status, branch, rank, rurality, service connected disabilities (percent), district, headache, chronic pain, medication-assisted treatment (recent), oncology, severe mental illness, depression, post-traumatic stress disorder, personality disorder, alcohol use disorder, OUD, other SUD, nicotine use disorder, anxiety, insomnia, congestive heart failure, perivascular disease, cardiac disease, stroke, diabetes mellitus (DM), DM with complications, epilepsy, neurologic disorder (no epilepsy), liver disease, chronic kidney disease, and death. CUD, cannabis use disorder; SCD, service-connected disability; TBI, traumatic brain injury; IRR, incidence rate ratio.

## Discussion

Compared with the No Dementia and No CUD group of veterans with confirmed TBI diagnosis, the highest annual total healthcare cost in VA and non-VA facilities was in veterans in the CUD only, which was associated with 1% less healthcare utilization. However, we observed the incidence rate of healthcare utilization in the dementia-related subgroups (Dementia only and Dementia and CUD groups) was 75% less than the No Dementia and No CUD group. Prior research has demonstrated that, compared with veterans without either TBI or dementia, veterans with TBI and dementia have the highest average annual costs of approximately USD$ 20,408, followed by the Dementia only (USD$ 4,822) and TBI only (USD$ 3,344) groups (3). Our findings suggest higher average healthcare costs in veterans with TBI and CUD compared with TBI and dementia. Cognitive dysfunction or impairment may reduce help-seeking intentions (45) and should be considered as a possible reason for lower dementia-related healthcare utilization. The significantly lower healthcare utilization without cost differences in veterans with a dementia diagnosis is consistent with higher dementia-related total costs that were found in long-term rehabilitation and domiciliary inpatient services (46). Despite the high prevalence and numerous associated adverse health consequences in individuals with CUD (47) and other individuals with substance use disorders (48, 49), other studies have found that veterans with CUD did not appear to seek treatment. In our study, compared with veterans with TBI but without dementia or CUD, veterans with TBI and CUD had the highest average annual healthcare costs, despite 1% lower healthcare utilization.

The timing of costs revealed the highest initial 5-year costs after TBI diagnosis were in the Dementia only group, which was driven by penetrating and moderate/severe TBI. Since the DoD healthcare costs were not included in our study, the immediate and expensive healthcare costs for penetrating and moderate/severe TBI are not reflected at the time of TBI. Other contributors to costs may include the persistence of TBI-related symptoms for more than 6 months post-injury (2) and the related needs of those veterans at high risk of various short- and long-term sequelae (50). Valuating the healthcare costs by subcategories in VA and non-VA facilities was beyond the focus of this study. However, relatively very high costs in the first 5 years (peak = $46,808 in year 4) could be explained by high-cost diagnostic tests, such as neuroimaging (51). The average time from TBI event to dementia diagnosis was 4.36 years for veterans in the dementia only group. Therefore, the highest initial in this group are less likely related to the prodromal phase of dementia where the veteran/family tries to find an answer to the cognitive difficulties, which needs further evaluation. While the burden of CUD costs is notable after 5 years following TBI, we did not observe extraordinary total healthcare costs in veterans with combined TBI, dementia, and CUD compared with those with dementia only in the first 5 years. These findings suggest that veterans with TBI and dementia only may be getting their healthcare needs met more quickly (i.e., in the first 5 years) while those with TBI, dementia, and CUD were not receiving sufficient initial care, resulting in higher healthcare costs after 5 years. Moreover, the absence of a high burden of healthcare costs in veterans with dementia and CUD in the short term (first 5 years) could be explained by the protective or regulatory effects of cannabis use (52, 53). The specific “causative” factors involved need further investigation, and it is possible that the antioxidant and anti-inflammatory properties of cannabidiol products (26) lead to a delay in seeking care in the first 5 years. Of note, after this initial period, costs of all types become much higher in those with dementia and CUD. Finally, dementia is a clinical diagnosis defined as at least two impaired mental functions that interfere with daily activities (54). Therefore, the documented dementia diagnosis may not represent all of the actual dementia cases. The sample sizes of veterans with TBI, CUD, and early onset dementia, diagnoses that have a high positive predictive value, were not large enough to replicate a sensitivity analysis from prior research (14). To overcome this limitation, we conducted a sensitivity analysis with confirmed dementia cases by identifying at least two dementia diagnoses. The sensitivity analysis only showed heterogeneity in the costs of dementia-related subgroups and warrants further evaluation.

This population-based study provides a broad view of the association of TBI, dementia, CUD, and VHA costs; however, as with any large database study, there were limitations. These results are limited to characteristics and conditions measured and stored in electronic health records (EHRs), which means that cannabis exposure information is limited to documented ICD codes in VA and DoD, which likely under-represents dosage and the chronicity of cannabis exposure. Of note, the EHR system in VHA allowed the inclusion of reliable study measures, such as the frequency of CUD and identification of the TBI index date relative to the documented development of CUD, strengthening our assessment of the associations between CUD, TBI, and dementia. While there are adequate techniques available to account for potential structural population differences in comorbidities and other expenditure-related factors to establish a proper cause-and-effect relationship (55), our study primarily relied on controlling for all measured covariates to focus on the excess burden of CUD in total healthcare costs and trajectories after TBI, providing a broader perspective on CUD costs for VHA. Nevertheless, in Supplementary Table S2, we provide an estimate of the potential extent of structural population differences based on dementia and CUD status. The ascertainment of the timing of TBI is problematic in our cohort and based on only the first time that a TBI diagnosis is noted in VA medical records. There is limited information on events such as lifetime TBI history and other variables such as type of brain injury (diffuse vs. focal), repetitive exposures, and mechanism of injury. Thus, TBIs reported here are not necessarily representative of service-connected injury (i.e., from deployed settings and/or related to military service) alone but may also include TBIs that occurred from a range of causes after leaving DoD (e.g., motor vehicle crashes, sports injuries, assault, and falls). While LIMBIC-CENC has engaged in an extensive effort to overcome this limitation and provide reliable TBI-related information, there are always certain limitations to Big Data analyses (i.e., optimization and empowerment of the data by aggregating information from different sources such as DoD and the diverse VA health system data sources; having an overpowered dataset; and using a dataset that was not originally designed to address the study question). Additionally, although private-sector care reimbursed by the VA is included in the analysis, private-sector care paid for by other third-party payers is not included.

Overall, healthcare costs in the TBI group that was identified as CUD only were higher than the dementia-related (Dementia only, Dementia, and CUD) groups. Lower healthcare utilization in the dementia-related groups could be explained by cognitive impairment and behavioral changes, limiting access to or perceived need for care in veterans suffering from dementia. The healthcare cost reduction after 14 years of TBI onset could also be explained by death in the dementia-related subgroups. A better appreciation of the timing and the types of services that are needed and/or accessed by these different subgroups of veterans is vital to optimize the availability and provision of the services. Given the constraints in overall resources across the VA system, it is important to assess the quality of supportive care in outpatient facilities by VA clinicians and administrators and to identify effective approaches to maximize cost-efficient strategies for veterans with TBI and at risk of dementia (11). The impact of the growing number of potential pharmacologic management options for dementia (56) and the extent to which such treatments may delay the need for healthcare services is unknown. Further investigation is needed to examine the impact of the timing of dementia and CUD diagnoses on veterans with TBI, with specific attention to the specific categories of inpatient and outpatient care in VA and community care facilities.

## Data availability statement

The datasets presented in this article are not readily available due to VA Regulations indicating data behind the firewall. Requests to access the datasets should be directed to: VINCI@VA.GOV.

## Ethics statement

The studies involving humans were approved by the University of Utah and Stanford University. The studies were conducted in accordance with the local legislation and institutional requirements. Written informed consent for participation was not required from the participants or the participants’ legal guardians/next of kin in accordance with the national legislation and institutional requirements.

## Author contributions

AE: Conceptualization, Data curation, Formal analysis, Funding acquisition, Investigation, Methodology, Resources, Software, Validation, Visualization, Writing – original draft, Writing – review & editing. TP: Writing – review & editing. MA: Data curation, Software, Formal analysis, Project administration, Writing – review & editing. CG: Project administration, Resources, Software, Writing – review & editing. AN: Writing – review & editing. MM: Project administration, Data curation, Software, Writing – review & editing. MP: Investigation, Supervision, Validation, Writing – review & editing. DC: Supervision, Writing – review & editing. CD-G: Supervision, Writing – review & editing.
