# Supplementary material for: The economic impact of cannabis use disorder and dementia diagnosis in veterans diagnosed with traumatic brain injury
Source: Front Neurol. 2024 Jan 12;14:1261144. doi: 10.3389/fneur.2023.1261144 (PMC10811113; doi:10.3389/fneur.2023.1261144)
Supplement: Supplementary file 1 [file Data_Sheet_1.docx]

Supplementary Material

**The Economic Impact of Cannabis Use Disorder and Dementia Diagnosis in Veterans Diagnosed with Traumatic Brain Injury.**

Aryan Esmaeili, MD, PhD (Corresponding Author),^1^ Terri K. Pogoda,^2,3^ Megan E. Amuan, MPH,^4,5^ Carla Garcia, MPH,^1^ Ariana Del Negro, BA,^1^ Maddy Myers,^4^ Mary Jo Pugh, PhD, RN,^4,5^ David Cifu, MD^6^, Clara Dismuke-Greer, PhD,^1^

^1^Health Economics Resource Center (HERC), Ci2i, VA Palo Alto Health Care System, Menlo Park, CA

^2^ Center for Healthcare Organization and Implementation Research, VA Boston Healthcare System, Boston, MA

^3^ Boston University School of Public Health, Boston, MA

^4^ Informatics, Decision-Enhancement, and Analytic Sciences Center of Innovation, VA Salt Lake City Health Care System, Salt Lake City, UT

^5^ Division of Epidemiology, Department of Internal Medicine, University of Utah School of Medicine, Salt Lake City, UT

^6^ Department of Physical Medicine and Rehabilitation, School of Medicine, Virginia Commonwealth University, Richmond, VA

**Corresponding Author’s Contact Information**

Aryan Esmaeili, 795 Willow Road, 152 MPD, Menlo Park, CA 94025, 843-814-0085, [aryan.esmaeili@va.gov](mailto:aryan.esmaeili@va.gov)

**Table S1: Variables Included in our study.**

| **Variables** | **Description** |
| --- | --- |
| **Outcomes (dementia and CUD)** | Dementia types included in the study: Creutzfeldt-Jakob Disease, Unspecified; Variant Creutzfeldt-Jakob Disease; Other Creutzfeldt-Jakob Disease; Progressive Multifocal Leukoencephalopathy; Gerstmann-Straussler-Scheinker Syndrome; Other Atypical Virus Infections Of Central Nervous System; Atypical Virus Infection Of Central Nervous System, Unspecified; Vascular Dementia Without Behavioral Disturbance; Vascular Dementia With Behavioral Disturbance; Dementia In Other Diseases Classified Elsewhere Without Behavioral Disturbance; Dementia In Other Diseases Classified Elsewhere With Behavioral Disturbance; Unspecified Dementia Without Behavioral Disturbance; Unspecified Dementia With Behavioral Disturbance; Alcohol Dependence With Alcohol-Induced Persisting Dementia; Alcohol Use, Unspecified With Alcohol-Induced Persisting Dementia; Sedative, Hypnotic Or Anxiolytic Dependence With Sedative, Hypnotic Or Anxiolytic-Induced Persisting Dementia; Sedative, Hypnotic Or Anxiolytic Use, Unspecified With Sedative, Hypnotic Or Anxiolytic-Induced Persisting Dementia; Inhalant Abuse With Inhalant-Induced Dementia; Inhalant Dependence With Inhalant-Induced Dementia; Inhalant Use, Unspecified With Inhalant-Induced Persisting Dementia; Other Psychoactive Substance Abuse With Psychoactive Substance-Induced Persisting Dementia; Other Psychoactive Substance Dependence With Psychoactive Substance-Induced Persisting Dementia; Other Psychoactive Substance Use, Unspecified With Psychoactive Substance-Induced Persisting Dementia; Progressive Supranuclear Ophthalmoplegia [Steele-Richardson-Olszewski]; Alzheimer's Disease With Early Onset; Alzheimer's Disease With Late Onset; Other Alzheimer's Disease; Alzheimer's Disease, Unspecified; Pick's Disease; Other Frontotemporal Dementia; Dementia With Lewy Bodies; Multi-System Degeneration Of The Autonomic Nervous System; Dementia In Other Diseases Classified Elsewhere Without Behavioral Disturbance/Human; Immunodeficiency Virus [Hiv] Disease; Dementia In Other Diseases Classified Elsewhere With Behavioral Disturbance/Human Immunodeficiency Virus [Hiv] Disease; Dementia In Other Diseases Classified Elsewhere Without Behavioral Disturbance/Huntington's Disease; Dementia In Other Diseases Classified Elsewhere With Behavioral Disturbance/Huntington's Disease; Dementia In Other Diseases Classified Elsewhere Without Behavioral Disturbance/Parkinson's Disease; Dementia In Other Diseases Classified Elsewhere With Behavioral Disturbance/Parkinson's Disease; Dementia In Other Diseases Classified Elsewhere Without Behavioral Disturbance/Idiopathic Normal Pressure Hydrocephalus; Dementia In Other Diseases Classified Elsewhere With Behavioral Disturbance/Idiopathic Normal Pressure Hydrocephalus.  Note: A diagnosis of dementia was identified using ICD-9/10 codes provided by Veterans Health Administration (VHA) geriatrics and extended care. To address a previously identified limitation of ICD codes not accurately capturing dementia in patients under the age of 65 (Salem et al., 2014;Marceaux et al., 2020), we expanded the dementia diagnosis definition in our cohort to also include cognitive impairment. |
|  | CUD: ICD-9 codes= Cannabis dependence (304.3), and Nondependent cannabis abuse (305.2), and ICD-10 codes= Cannabis-related disorders (F12). |
| **Comorbid Conditions after TBI index date, using a conceptual framework (Nuckols et al., 2013)** | Demographic and military characteristics:  *Verified by LIMBIC (VADIR): Sex, Age at the time of TBI, TBI severity, branch, rank, Rurality, service connected disabilities (percent)*  *Using VA CDW: Race, Education, Marital status, District, Death* |
|  | Clinical and mental health characteristics:  Selected Elixhauser co-morbidities were identified as categorical variables (yes/no) based on ICD-9 or ICD-10 codes identified as: congestive heart failure, cardiac arrhythmias, peripheral vascular disorders, uncomplicated diabetes, complicated diabetes, depression, liver disease, Chronic Kidney Disease, alcohol abuse, drug abuse, and insomnia, sleep Apnea (Elixhauser et al., 1998).  Post-traumatic stress disorder (PTSD), anxiety, (Kennedy, 2022).  Headache, Stroke, Epilepsy, Neurologic disorder (Other than epilepsy) (Hai et al., 2023),  Chronic Pain (other than headache): including IBS, Back pain, Myalgia/Myositis, Arthropathy/Tendinopathy, Osteoarthritis, Crystal Arthropathies, Neck/Spine pain, Neuropathy, SCI, Menstrual Disorders, Vulvodynia, Interstitial Cystitis, Endometriosis.  Oncology and Hospice: identified using clinical stop codes. Veterans with a history of >2 visits to oncology clinics are considered patients who treat for cancer.  Medication-assisted treatment (MAT): using algorithm provided by Pharmacy Benefits Management Services (PBM). If patient with history of OUD is on the following prescription or conditions: [a. Buprenorphine, b. Naltrexone tablet (for outpatient prescriptions, and CPRS orderable items), c. Naltrexone injection (for outpatient prescriptions, CPRS orderable items, and outpatient procedures CPT Code 'J2315'), d. Methadone] OR e. Outpatient visit to stop code for Visits to Opioid Treatment Program (OTP) ‘523’. |
| **Covariates in the adjusted model** | *The covariates included in the adjusted model: Year with TBI, gender, age at the time of TBI, TBI severity, race, education, marital status, branch, rank, Rurality, service connected disabilities (percent), District, Headache, ChronicPain, MAT (recent), Oncology, SMI, Depression, PTSD, Personality Disorder, Alcohol Use Disorder, Opioid Use Disorder, Other SUD, Nicotine Use disorder, anxiety, insomnia, CHF, Perivascular disease, Cardiac disease, Stroke, DM, DM with complications, Epilepsy, Neurologic disorder (NoEpilepsy), Liver Disease, CKD, and death.* |

Table S2- Demographic and Clinical Characteristics of Veterans with a history of TBI by dementia diagnosis and CUD status (N=387,770). The Standardized Mean Difference calculated the potential clinical and structural population differences between the Dementia and Non-dementia and CUD and Non-CUD groups.

|  | No Dementia & No CUD  N (%) | Dementia Only  N (%) | CUD Only  N (%) | Dementia & CUD  N (%) | Total  N (%) | The Standardized Mean Difference Dementia | The Standardized Mean Difference CUD |
| --- | --- | --- | --- | --- | --- | --- | --- |
| Overall | 341324 (88.02) | 4572 (1.18) | 40873 (10.54) | 1001 (0.26) | 387,770 (100) |  |  |
| Male | 300291 (87.98) | 4078 (89.2) | 37319 (91.3) | 924 (92.31) | 342612 (88.35) | -0.045 | **-0.110** |
| Age≥65 | 2228 (0.65) | 316 (6.91) | 27 (0.07) | 4 (0.4) | 2575 (0.66) | **-0.297** | **0.104** |
| **Race and Ethnicity** |  |  |  |  |  | 0.007 | -0.051 |
| White | 217674 (63.77) | 2887 (63.15) | 25167 (61.57) | 629 (62.84) | 246357 (63.53) |  |  |
| Black/African American | 54081 (15.84) | 800 (17.5) | 7042 (17.23) | 169 (16.88) | 62092 (16.01) |  |  |
| Hispanic or Latino | 35339 (10.35) | 432 (9.45) | 3543 (8.67) | 86 (8.59) | 39400 (10.16) |  |  |
| Other | 32957 (9.66) | 446 (9.76) | 5036 (12.32) | 114 (11.39) | 38553 (9.94) |  |  |
| Unknown | 1273 (0.37) | 7 (0.15) | 85 (0.21) | 3 (0.3) | 1368 (0.35) |  |  |
| **Education** |  |  |  |  |  | **0.220** | **-0.355** |
| College and above | 81834 (23.98) | 1679 (36.72) | 4349 (10.64) | 142 (14.19) | 88004 (22.69) |  |  |
| High School and Less | 258910 (75.85) | 2873 (62.84) | 36474 (89.24) | 856 (85.51) | 299113 (77.14) |  |  |
| Unknown | 580 (0.17) | 20 (0.44) | 50 (0.12) | 3 (0.3) | 653 (0.17) |  |  |
| **Marital Status** |  |  |  |  |  | **-0.129** | **0.356** |
| Unmarried | 161077 (47.19) | 1763 (38.56) | 26386 (64.56) | 614 (61.34) | 189840 (48.96) |  |  |
| Married | 180067 (52.76) | 2808 (61.42) | 14471 (35.4) | 386 (38.56) | 197732 (50.99) |  |  |
| Unknown | 180 (0.05) | 1 (0.02) | 16 (0.04) | 1 (0.1) | 198 (0.05) |  |  |
| **Military Branch** |  |  |  |  |  | 0.050 | 0.024 |
| Air Force | 33398 (9.78) | 686 (15) | 2399 (5.87) | 88 (8.79) | 36571 (9.43) |  |  |
| Army | 204047 (59.78) | 2615 (57.2) | 27442 (67.14) | 658 (65.73) | 234762 (60.54) |  |  |
| Marines | 61003 (17.87) | 551 (12.05) | 6822 (16.69) | 135 (13.49) | 68511 (17.67) |  |  |
| Navy/Coast Guard | 42725 (12.52) | 708 (15.49) | 4208 (10.3) | 120 (11.99) | 47761 (12.32) |  |  |
| Other | 151 (0.04) | 12 (0.26) | 2 (0) | 0 (0) | 165 (0.04) |  |  |
| **Rank** |  |  |  |  |  | **-0.188** | **0.268** |
| Enlisted | 317218 (92.95) | 3916 (85.65) | 40288 (98.57) | 974 (97.3) | 362396 (93.46) |  |  |
| Officer | 20407 (5.98) | 562 (12.29) | 484 (1.18) | 24 (2.4) | 21477 (5.54) |  |  |
| Warrant | 3670 (1.08) | 94 (2.06) | 99 (0.24) | 3 (0.3) | 3866 (1) |  |  |
| **Rurality** |  |  |  |  |  | 0.003 | -0.015 |
| Rural | 108255 (31.72) | 1455 (31.82) | 12632 (30.91) | 309 (30.87) | 122651 (31.63) |  |  |
| Urban | 231902 (67.94) | 3109 (68) | 28151 (68.87) | 689 (68.83) | 263851 (68.04) |  |  |
| Unknown | 1167 (0.34) | 8 (0.17) | 90 (0.22) | 3 (0.3) | 1268 (0.33) |  |  |
| VA SCD Percent (0) | 38954 (11.41) | 665 (14.55) | 4928 (12.06) | 132 (13.19) | 44679 (11.52) | 0.027 | -0.021 |
| 10 to 40 | 24126 (7.07) | 157 (3.43) | 1821 (4.46) | 10 (1) | 26114 (6.73) |  |  |
| ≥50 | 278244 (81.52) | 3750 (82.02) | 34124 (83.49) | 859 (85.81) | 316977 (81.74) |  |  |
| **District** |  |  |  |  |  | 0.067 | -0.029 |
| North Atlantic | 70682 (20.71) | 956 (20.91) | 8177 (20.01) | 188 (18.78) | 80003 (20.63) |  |  |
| Southeast | 66029 (19.35) | 1090 (23.84) | 7634 (18.68) | 198 (19.78) | 74951 (19.33) |  |  |
| Midwest | 69456 (20.35) | 854 (18.68) | 8524 (20.85) | 177 (17.68) | 79011 (20.38) |  |  |
| Continental | 72831 (21.34) | 1042 (22.79) | 8722 (21.34) | 260 (25.97) | 82855 (21.37) |  |  |
| Pacific | 62318 (18.26) | 630 (13.78) | 7816 (19.12) | 178 (17.78) | 70942 (18.3) |  |  |
| **Comorbid Conditions** |  |  |  |  |  |  |  |
| Headache | 194574 (57.01) | 3178 (69.51) | 24497 (59.93) | 729 (72.83) | 222978 (57.5) | **-0.268** | -0.062 |
| Other Chronic Pain | 309908 (90.8) | 4386 (95.93) | 38233 (93.54) | 964 (96.3) | 353491 (91.16) | **-0.201** | **-0.103** |
| MAT (recent) | 18957 (5.55) | 335 (7.33) | 11333 (27.73) | 317 (31.67) | 30942 (7.98) | **-0.127** | **-0.625** |
| Oncology | 4257 (1.25) | 152 (3.32) | 611 (1.49) | 20 (2) | 5040 (1.3) | **-0.124** | -0.020 |
| SMI | 80657 (23.63) | 2184 (47.77) | 24516 (59.98) | 786 (78.52) | 108143 (27.89) | **-0.544** | **-0.795** |
| Depression | 187134 (54.83) | 3614 (79.05) | 34257 (83.81) | 940 (93.91) | 225945 (58.27) | **-0.537** | **-0.662** |
| PTSD | 222236 (65.11) | 3058 (66.89) | 35512 (86.88) | 895 (89.41) | 261701 (67.49) | -0.076 | **-0.529** |
| Personality Disorder | 15343 (4.5) | 474 (10.37) | 9748 (23.85) | 375 (37.46) | 25940 (6.69) | **-0.281** | **-0.582** |
| Alcohol Use Disorder | 120007 (35.16) | 1967 (43.02) | 33786 (82.66) | 886 (88.51) | 156646 (40.4) | **-0.221** | **-1.104** |
| OUD | 20292 (5.95) | 532 (11.64) | 18187 (44.5) | 533 (53.25) | 39544 (10.2) | **-0.258** | **-0.993** |
| Other Drug Use Disorder | 23526 (6.89) | 624 (13.65) | 26766 (65.49) | 790 (78.92) | 51706 (13.33) | **-0.313** | **-1.545** |
| Nicotine Use Disorder | 91915 (26.93) | 1494 (32.68) | 23006 (56.29) | 676 (67.53) | 117091 (30.2) | **-0.187** | **-0.628** |
| Anxiety | 171582 (50.27) | 3098 (67.76) | 30976 (75.79) | 867 (86.61) | 206523 (53.26) | **-0.381** | **-0.550** |
| Insomnia | 114882 (33.66) | 2246 (49.13) | 16147 (39.51) | 557 (55.64) | 133832 (34.51) | **-0.328** | **-0.125** |
| CHF | 6880 (2.02) | 504 (11.02) | 794 (1.94) | 70 (6.99) | 8248 (2.13) | **-0.350** | 0.005 |
| Peripheral vascular Disease | 12441 (3.64) | 840 (18.37) | 1152 (2.82) | 93 (9.29) | 14526 (3.75) | **-0.447** | 0.048 |
| Cardiac Disease | 48580 (14.23) | 1618 (35.39) | 7806 (19.1) | 372 (37.16) | 58376 (15.05) | **-0.497** | **-0.134** |
| Stroke | 12634 (3.7) | 1145 (25.04) | 1538 (3.76) | 187 (18.68) | 15504 (4) | **-0.612** | -0.007 |
| DM | 37526 (10.99) | 1219 (26.66) | 2985 (7.3) | 178 (17.78) | 41908 (10.81) | **-0.385** | **0.125** |
| Diabetes with chronic complication | 21038 (6.16) | 795 (17.39) | 1600 (3.91) | 99 (9.89) | 23532 (6.07) | **-0.328** | **0.102** |
| Epilepsy | 85489 (25.05) | 2186 (47.81) | 19406 (47.48) | 664 (66.33) | 107745 (27.79) | **-0.500** | **-0.482** |
| Other Neurologic Disorders (No Epilepsy) | 6515 (1.91) | 889 (19.44) | 992 (2.43) | 139 (13.89) | 8535 (2.2) | **-0.566** | -0.036 |
| Liver Disease | 12905 (3.78) | 325 (7.11) | 1810 (4.43) | 83 (8.29) | 15123 (3.9) | **-0.152** | -0.035 |
| CKD | 6233 (1.83) | 302 (6.61) | 801 (1.96) | 43 (4.3) | 7379 (1.9) | **-0.223** | -0.009 |
| Death | 10068 (2.95) | 647 (14.15) | 2561 (6.27) | 119 (11.89) | 13395 (3.45) | **-0.381** | **-0.156** |
| **TBI Severity and Evidence of TBI** |  |  |  |  |  | -0.056 | 0.087 |
| Mild | 223,940 (65.61) | 2,304 (50.39) | 27,358 (66.93) | 539 (53.85) | 254,141 (65.54) |  |  |
| Moderate/Severe | 44421 (13.01) | 955 (20.89) | 6069 (14.85) | 233 (23.28) | 51678 (13.33) |  |  |
| Penetrating | 11582 (3.39) | 702 (15.35) | 1450 (3.55) | 137 (13.69) | 13871 (3.58) |  |  |
| Unclassified | 61381 (17.98) | 611 (13.36) | 5996 (14.67) | 92 (9.19) | 68080 (17.56) |  |  |
| *Abbreviations: CUD= Cannabis Use Disorder, SCD=Service Connected Disability, TBI= Traumatic Brain Injury, MAT= Medication-assisted treatment, CHF= Congestive Heart Failure, CKD= Chronic Kidney Disease, PTSD= Post Traumatic Stress Disorder, SMI= Severe Mental Illness, DM= Diabetes Mellitus.*  *The absolute value of standardized mean differences (SMDs) greater than 0.1 were interpreted as an important difference between groups.(Austin, 2009b;Austin, 2009a;Ranganathan et al., 2015)* | | | | | | | |

Table S3- The Average of the Total Healthcare Costs, First Year, First 5 Years, and First 15 Years after TBI, By TBI and CUD Status.

|  | Control  Mean (SEM) | Dementia Only  Mean (SEM) | CUD Only  Mean (SEM) | Dementia & CUD  Mean (SEM) | Total Mean (SEM) | p* |
| --- | --- | --- | --- | --- | --- | --- |
| **Total Healthcare costs, first year after TBI** | | | | | | |
| Mild | 71.89 (42.55) | 153.1 (114.81) | 16.68 (9.91) | 31.34 (31.34) | 66.59 (37.53) | 0.0001 |
| Moderate/Severe | 341.18 (159.08) | 1670.8 (1047.22) | 22.82 (6.67) | 1226.79 (683.09) | 332.35 (138.14) | 0.0001 |
| Penetrating | 979.17 (700.45) | 2537.1 (2505.98) | 12.93 (6.21) | 18.05 (18.05) | 947.52 (598.45) | 0.0034 |
| Unclassified | 15.84 (6.43) | 8.45 (7.18) | 8.85 (7.18) | 0 (0) | 15.14 (5.83) | 0.2404 |
| **Total Healthcare costs, first 5 years after TBI** | | | | | | |
| Mild | 591.61 (48.35) | 884.52 (248.77) | 1013.89 (202) | 2098.38 (751.14) | 642.92 (47.91) | 0.0001 |
| Moderate/Severe | 3738.21 (583.96) | 61360.05 (27663.21) | 1165.62 (214.3) | 4935.67 (1218.77) | 4506.33 (717.04) | 0.0001 |
| Penetrating | 4725.81 (640.2) | 30497.62 (12633.98) | 1073.77 (204.71) | 1902.21 (685.62) | 5620.45 (833.94) | 0.0001 |
| Unclassified | 311.26 (44.64) | 1120.99 (366.76) | 283.88 (40.31) | 278.88 (140.39) | 316.07 (40.54) | 0.0001 |
| **Total Healthcare costs, first 15 years after TBI** | | | | | | |
| Mild | 4334.59 (19.37) | 7317.99 (241.31) | 12116.27 (95.62) | 17497.78 (857.82) | 5227.24 (20.17) | 0.0001 |
| Moderate/Severe | 6743.91 (200.14) | 37010.47 (9300.22) | 14225.51 (190.99) | 20140.21 (1396.08) | 8242.26 (244.34) | 0.0001 |
| Penetrating | 6449.03 (246.41) | 27888.67 (4372.08) | 12488.49 (363.84) | 19918.63 (2243.28) | 8298.44 (305.53) | 0.0001 |
| Unclassified | 2704.35 (22.56) | 5591.03 (479.13) | 8014.33 (122.41) | 17863.35 (2016.9) | 3218.41 (23.63) | 0.0001 |
| *Abbreviations: CUD= Cannabis Use Disorder, TBI= Traumatic Brain Injury, SEM= Standard Error of the Mean.*  ** Using Kruskal–Wallis test* | | | | | | |

Appendix References:

Austin, P.C. (2009a). Balance diagnostics for comparing the distribution of baseline covariates between treatment groups in propensity-score matched samples. *Stat Med* 28**,** 3083-3107.

Austin, P.C. (2009b). Using the Standardized Difference to Compare the Prevalence of a Binary Variable Between Two Groups in Observational Research. *Communications in Statistics - Simulation and Computation* 38**,** 1228-1234.

Elixhauser, A., Steiner, C., Harris, D.R., and Coffey, R.M. (1998). Comorbidity measures for use with administrative data. *Med Care* 36**,** 8-27.

Hai, T., Agimi, Y., and Stout, K. (2023). Prevalence of Comorbidities in Active and Reserve Service Members Pre and Post Traumatic Brain Injury, 2017-2019. *Mil Med* 188**,** e270-e277.

Marceaux, J.C., Soble, J.R., O'rourke, J.J.F., Swan, A.A., Wells, M., Amuan, M., Sagiraju, H.K.R., Eapen, B.C., and Pugh, M.J. (2020). Validity of early-onset dementia diagnoses in VA electronic medical record administrative data. *Clin Neuropsychol* 34**,** 1175-1189.

Nuckols, T.K., Escarce, J.J., and Asch, S.M. (2013). The effects of quality of care on costs: a conceptual framework. *Milbank Q* 91**,** 316-353.

Ranganathan, P., Pramesh, C.S., and Buyse, M. (2015). Common pitfalls in statistical analysis: Clinical versus statistical significance. *Perspect Clin Res* 6**,** 169-170.

Salem, L.C., Andersen, B.B., Nielsen, T.R., Stokholm, J., Jorgensen, M.B., and Waldemar, G. (2014). Inadequate diagnostic evaluation in young patients registered with a diagnosis of dementia: a nationwide register-based study. *Dement Geriatr Cogn Dis Extra* 4**,** 31-44.
